# On the Application of Bandages to the Lower Extremities in Uterine Hemorrhage

**Published:** 1848-01

**Authors:** 


The editors of the St. Louis Medical and Surgical Journal make the following remarks in the November number of their Journal.

"*On the Application of Bandages to the lower extremities in Uterine Hemorrhagy.*—We penned an article some months ago, in which we deduced from admitted principles the utility of this means of treating flooding. We had not at that time submitted the remedy to the test of experience. An opportunity was shortly afterwards afforded us by the following case: Mrs. S., of previously good health and robust constitution, had been delivered one hour, when we were called to see her on account of an alarming hemorrhagy. We found her pale, almost pulseless, sighing frequently, the head hanging over the edge of the bed—there were smart intermittent pains. On examination, the uterine tumor was found to be large and somewhat hard, a firm clot was protruding from the vagina—the external bleeding had not been very considerable. Ergot was sent for, but before it could be procured, firm clots, to the amount of a quart or more, were expelled, together with some uncoagulated blood. The state of the patient now became extremely alarming, for though the uterus was tolerably firmly contracted, and a further loss of blood, to any great degree, was not to be apprehended, the system was *already* in a state of prostration, from which we feared it would be difficult to arouse it. The pulse could scarcely be felt—the face of a pale leaden hue, the breathing irregular. Stimulants were given; cold applications to the hypogastrum were made with but little apparent effect. Then it was that the roller was resorted to. A sheet was immediately torn into tolerably wide bands, and these were applied to both legs, commencing at the feet, and ending near the hip joint. The improvement of the patient's condition was soon evident, and in a half hour we felt that we might leave her without apprehending danger.

"Called again in six hours—patient much better. At this visit she passed the following eulogy on the roller: "As soon as it was applied, I felt that I was strengthened afresh, and my faintly feelings revived." Though aware that the roller had been eminently serviceable in the case, this confession of the patient tended no little to strengthen our faith in this highly rational method. It is not necessary to discuss the mode in which it acts, as every one will see that it is by supplying the general system with blood from the legs. Is such a mode of managing these cases not well calculated as a substitute for the difficult and dubious operation of transfusion?

"Since the publication of the article referred to by our colleague, in the number of this journal for July last, we have had an opportunity of trying the effect of bandages to the lower extremities in one severe case of uterine hemorrhage, which we subjoin in further corroberation of his views. The patient, Mrs. W., rather a weak and delicate woman, whose health has suffered from the effect of former miscarriages. On the 16th of September we were called to her at night, and found her with severe intermittent pains in the lower part of the abdomen, and a profuse sanguinous discharge from the vagina. On examination found the os uteri dilated so as to receive the end of the index finger. She supposed herself in the third month of pregnancy, and attributed her present mishap to a fall which she had some days before. Ordered one grain of opium, three of acetate of lead, and two of camphor, to be administered every half hour, hour, or two hours, according to the urgency of the symptoms, cold drinks, absolute rest with the head almost on a level with the body, and if necessary, cold applications to the hypogastric regions. In a few hours the severity of the symptoms subsided, and we left her for the night. On the 17th, 18th, and 19th, she gradually grew better, and our visits were discontinued, with strict injunctions as to rest, &c., &c. On the 30th we were again called to see her, and found that the pains had returned with great severity, and that the flooding was very profuse, coming away not in clots, but warm from the uterus. Pulse exceedingly feeble and frequent, countenance palid, and complaining of general uneasiness. The most active remedies were now resorted to, including ergot (it being evident that the placenta was detached, and that the fœtus must pass off,) and the bandages. The latter were applied, about three inches wide, from the toes to near the groin of each leg. A few moments after the application, the pulse was carefully examined, and our impression was that it exhibited a decided improvement, when the patient remarked that the bandages made her feel "very comfortable." The case subsequently did well. We would not of course pretend to pass on the merits of any remedy upon the evidence afforded by a single case, but the idea is novel, and, as we think, plausible—and it is worth while to repeat the experiments. It is not unreasonable to suppose that six or eight ounces of blood may in this way be supplied to the general system, and to that extent the drooping powers of life sustained, while other remedies are brought to bear. But even admitting that it does no good in the way of supplying blood, yet if it makes the patient feel comfortable, that of itself is a sufficient reason for the use of the bandages as proposed."

